# Efficacy of ivabradine for heart failure: A protocol for a systematic review of randomized controlled trial

**DOI:** 10.1097/MD.0000000000015075

**Published:** 2019-04-05

**Authors:** En-zhong Xue, Ming-hui Zhang, Chun-li Liu

**Affiliations:** a Department of Cardiology; b Department of Endocrinology, The People's Hospital of Yan’an, Yan’an, China.

**Keywords:** efficacy, heart failure, ivabradine, safety, systematic review

## Abstract

**Background:**

Previous clinical trials have reported that ivabradine can effectively treat heart failure (HF). However, no systematic review has explored its efficacy and safety for HF. This systematic review will aim to evaluate the efficacy and safety of ivabradine for the treatment of patients with HF.

**Methods:**

We will search the literature from the following electronic databases from inception to the January 31, 2019: Cochrane Central Register of Controlled Trials, EMBASE, MEDILINE, Web of Science, Cumulative Index to Nursing and Allied Health Literature, Allied and Complementary Medicine Database, Chinese Biomedical Literature Database, China National Knowledge Infrastructure, VIP Information, and Wanfang Data. All randomized controlled trials (RCTs) of ivabradine for HF will be fully considered for inclusion without any restrictions. Additionally, grey literature including clinical trial registries, dissertations, and reference lists of included studies, conference abstracts will also be searched. Two researchers will review these literatures, extract data, and assess risk of bias of included RCTs separately. Data will be pooled by either fixed-effects model or random-effects model, and meta-analysis will be conducted if it is appropriate.

**Results:**

The primary outcome is all-cause mortality. The secondary outcomes comprise of change in body weight, urine output, change in serum sodium, and all adverse events.

**Conclusions:**

The results of this study will summary provide up-to-dated evidence for assessing the efficacy and safety of ivabradine for HF.

**Ethics and Dissemination:**

It is not necessary to acquire ethical approval for this systematic review, because no individual patient data will be used in this study. The results of this systematic review will be published through peer-reviewed journals.

**Systematic review registration:**

PROSPERO CRD42019120814.

## 1 Introduction

Heart failure (HF) is the one of the most common cardiovascular disorders.^[1–3]^ It is estimated that 1 in 5 people will develop HF in their lifetime.^[4,5]^ Its prevalence is estimated at 2% in the western countries and its incidence approaches 5 to 10 per 1000 persons each year.^[6–8]^ In addition, its prevalence is 7% in 75 to 84 years old and over 10% in people older than 85 years old.^[9–11]^ Moreover, its 5-year age-adjusted mortality rates after onset are estimated to be 50% in men and 46% in women.^[12,13]^ The treatment for HF often brings very heavy economic burden for patients and their families.^[14]^

Previous clinical studies have reported that ivabradine can effectively treat patients with HF.^[15–27]^ However, no systematic review has been conducted to assess its efficacy and safety for HF according to the current available published trials. Thus, in the present study, we will systematically investigate the efficacy and safety of ivabradine for patients with HF.

## 2 Methods and analysis

### 2.1 Study registration

The systematic review protocol has been registered on PROSPERO (CRD42019120814). It is designed follows the Preferred Reporting Items for Systematic Reviews and Meta-Analysis Protocol statement guidelines.^[28]^

### 2.2 Inclusion criteria for study selection

#### 2.2.1 Types of studies

This study will include randomized controlled trials (RCTs) alone of ivabradine for HF without any language or publication status restrictions. Non-clinical trials, uncontrolled trials, case studies, crossover studies, non-RCTs, and quasi-RCTs will all be not considered for inclusion.

#### 2.2.2 Types of participants

Participants with a diagnosis of HF will be considered without restrictions of their age, sex, race, education, or economic status.

#### 2.2.3 Types of interventions

##### 2.2.3.1 Experimental interventions

We will include any forms of ivabradine alone in the experimental group. However, we will include the studies using combined treatments of ivabradine and any other therapies as an experimental intervention.

##### 2.2.3.2 Control interventions

Interventions in the control group can be any treatments, except the ivabradine.

##### 2.2.3.3 Type of outcome measurements

The primary outcome is all-cause mortality. The secondary outcomes comprise of change in body weight, urine output, change in serum sodium, and all adverse events.

### 2.3 Literature search

The primary source of information will be the following databases from inception to the January 31, 2019: Cochrane Central Register of Controlled Trials (CENTRAL), EMBASE, MEDILINE, Web of Science, Cumulative Index to Nursing and Allied Health Literature, Allied and Complementary Medicine Database, Chinese Biomedical Literature Database, China National Knowledge Infrastructure, VIP Information, and Wanfang Data without any language restrictions. The secondary sources include Google scholar, clinical trial registration, reference lists of eligible studies, and conference proceedings.

The keywords will be combined with terms associated with “ivabradine,” “heart failure,” “cardiac failure,” ‘cardiac insufficient,” “randomized controlled trial,” “randomized,” “trial,” and “clinical trial” for searching ivabradine for HF. The detailed search strategy of CENTRAL is showed in Table 1. Similar strategies will be used to other databases.

**Table 1:**
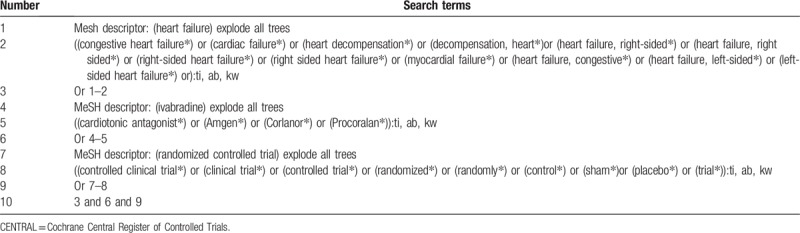
Search strategy applied in CENTRAL database.

### 2.4 Study selection, data extraction, and management

#### 2.4.1 Study selection

**Figure 1:**
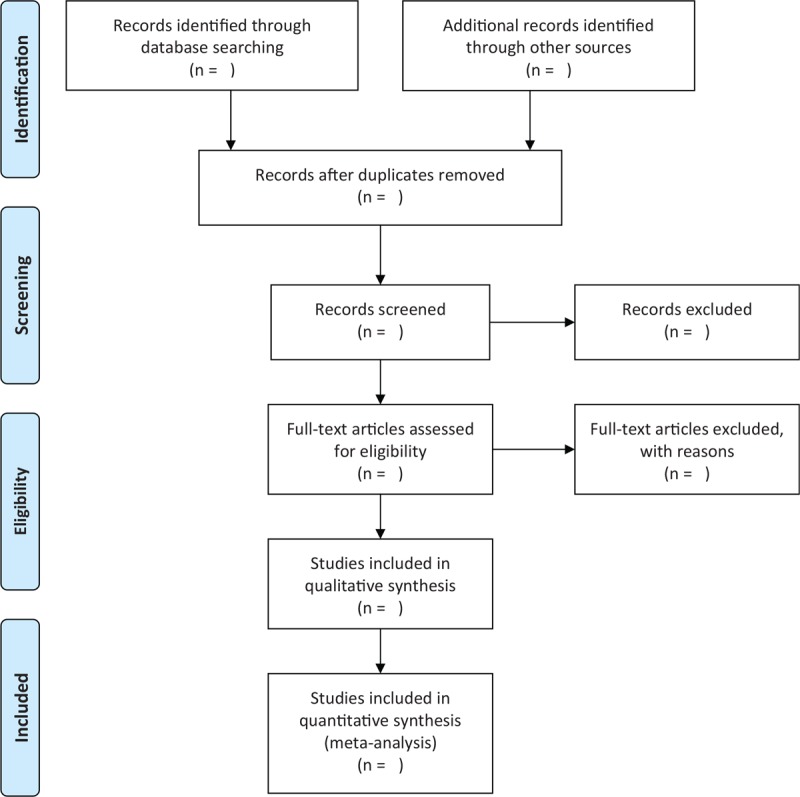
Process of study selection.

Two researchers will independently select studies according to the title or abstract, as well as the full text only if insufficient to decide about the inclusion criteria through the abstract. Any divergences will be dealt with by a third researcher through discussion. The process of study selection is showed in Fig. 1.

#### 2.4.2 Data extraction and management

A standardized form will be used to extract all data by 2 researchers independently. The form will include first author, year of publication, location, study design, interventions, and outcome measurements and reporting. Any disagreements of data extraction between 2 researchers will be settled down by a third researcher through discussion. Any insufficient information or missing data will be required by contacting the authors of the primary included RCTs.

### 2.5 Strategy for data synthesis

#### 2.5.1 Risk of bias assessment

This study will use Cochrane risk of bias tool to evaluate the methodological quality for each enrolled study. The risk of bias will be assessed by 2 independent researchers, and any disagreements will be resolved by discussion with other researchers.

#### 2.5.2 Measurement of treatment effect

As for dichotomous data, we will apply risk ratio with 95% confidence intervals (CIs). As for continuous data, we will utilize mean difference or standardized mean difference with 95% CIs.

#### 2.5.3 Data synthesis

The heterogeneity will be tested through *I*^2^ test. If *I*^2^ is <50%, a fixed-effect model will be used to synthesize the data, and meta-analysis will be conducted by using RevMan 5.3 software (Cochrane Community, London, UK). If *I*^2^ is more than 50%, a random-effect model will be applied to synthesize the data, and subgroup analysis will be performed according to the different interventions, controls, and outcome measurement tools. If the heterogeneity is still significant after subgroup analysis, data will not be synthesized, and a narrative summary will be presented. Additionally, sensitivity analysis will also be conducted to eliminate the impact of low quality studies.

#### 2.5.4 Reporting biases

Regarding for reporting bias, we will evaluate the symmetry of funnel plots >10 eligible studies are included for meta-analysis conduction.^[29]^

## 3 Discussion

This systematic review will investigate the efficacy and safety of ivabradine for the treatment of HF. To our best knowledge, this systematic review is the first study to assess the efficacy of ivabradine in patients with HF. This systematic review will pool the outcome data and will provide the current evidence on the efficacy and safety of ivabradine for the treatment of HF. This review may present solid data and robust evidence to the clinicians, future research protocols, as well as for the health policy makers.

## Author contributions

**Conceptualization:** En-zhong Xue, Ming-hui Zhang, Chun-li Liu.

**Data curation:** En-zhong Xue, Chun-li Liu.

**Formal analysis:** En-zhong Xue, Ming-hui Zhang.

**Funding acquisition:** Chun-li Liu.

**Investigation:** Chun-li Liu.

**Methodology:** Ming-hui Zhang.

**Project administration:** Chun-li Liu.

**Resources:** En-zhong Xue, Ming-hui Zhang.

**Software:** En-zhong Xue, Ming-hui Zhang.

**Supervision:** Chun-li Liu.

**Validation:** En-zhong Xue, Chun-li Liu.

**Visualization:** Ming-hui Zhang, Chun-li Liu.

**Writing – Original Draft:** En-zhong Xue, Ming-hui Zhang, Chun-li Liu.

**Writing – Review & Editing:** En-zhong Xue, Ming-hui Zhang, Chun-li Liu.
